# Correction: A *Ctnnb1* enhancer transcriptionally regulates Wnt signaling dosage to balance homeostasis and tumorigenesis of intestinal epithelia

**DOI:** 10.7554/eLife.113472

**Published:** 2026-10-14

**Authors:** Xiaojiao Hua, Chen Zhao, Jianbo Tian, Junbao Wang, Xiaoping Miao, Gen Zheng, Min Wu, Mei Ye, Ying Liu, Yan Zhou

**Keywords:** Mouse

Hua X, Zhao C, Tian J, Wang J, Miao X, Zheng G, Wu M, Ye M, Liu Y, Zhou Y. 2024. A *Ctnnb1* enhancer transcriptionally regulates Wnt signaling dosage to balance homeostasis and tumorigenesis of intestinal epithelia. *eLife* **13**:RP98238. doi: 10.7554/eLife.98238.

Published 25 September 2024

1. Incorrect representative immunofluorescence image for the *Ctnnb1^Δi.enh^* group in Figure 2L

For the immunofluorescence image representing the *Ctnnb1^Δi.enh^* group in Figure 2L we in error used an image from the *H11^i.enh^* group.

The *H11^i.enh^* and *Ctnnb1^Δi.enh^* samples were processed and imaged in parallel, and the error was introduced during manual preparation of Figure 2 when the image files were handled together. We now correct Figure 2L to show the correct representative *Ctnnb1^Δi.enh^* image.

2. Overlapping fields used for EdU-positive cell quantification in the *H11^i.enh^* group in Figure 2L

Within the *H11^i.enh^* group, two cropped fields used for quantification (21#-C and 29#-C) were mistakenly taken from partially overlapping intestinal epithelium crops from the same animals (21#-A and 29#-B, respectively).

For both the *H11^i.enh^* and *Ctnnb1^Δi.enh^* groups, six mice per group were used. After EdU injection, three intestinal fields were imaged per mouse, and the average number of EdU-positive cells per crypt–villus unit across the three fields was taken as the final value for that mouse. When retracing the original data, we found that two of the fields used for quantification in the *H11^i.enh^* group (21#-C and 29#-C) partially overlapped with other fields imaged from the same animals (21#-A and 29#-B), respectively. We have therefore excluded the EdU-positive cell counts derived from fields 21#-C and 29#-C of the *H11^i.enh^* group and repeated the quantification using the remaining non-overlapping fields for the two mice concerned; the number of animals analyzed per group (n=6) is unchanged, and the re-analyzed results are consistent with the conclusions reported in the paper (the correction process is shown in the figure below). The recalculation changes the mean number of EdU-positive cells per crypt–villus unit in the control group from 41.83±6.68–41.81±6.12, and the decrease reported in the article from 21.90% to 21.86%; the value of 21.9% given in the main text is therefore unchanged at the precision reported.

**Figure 3:**
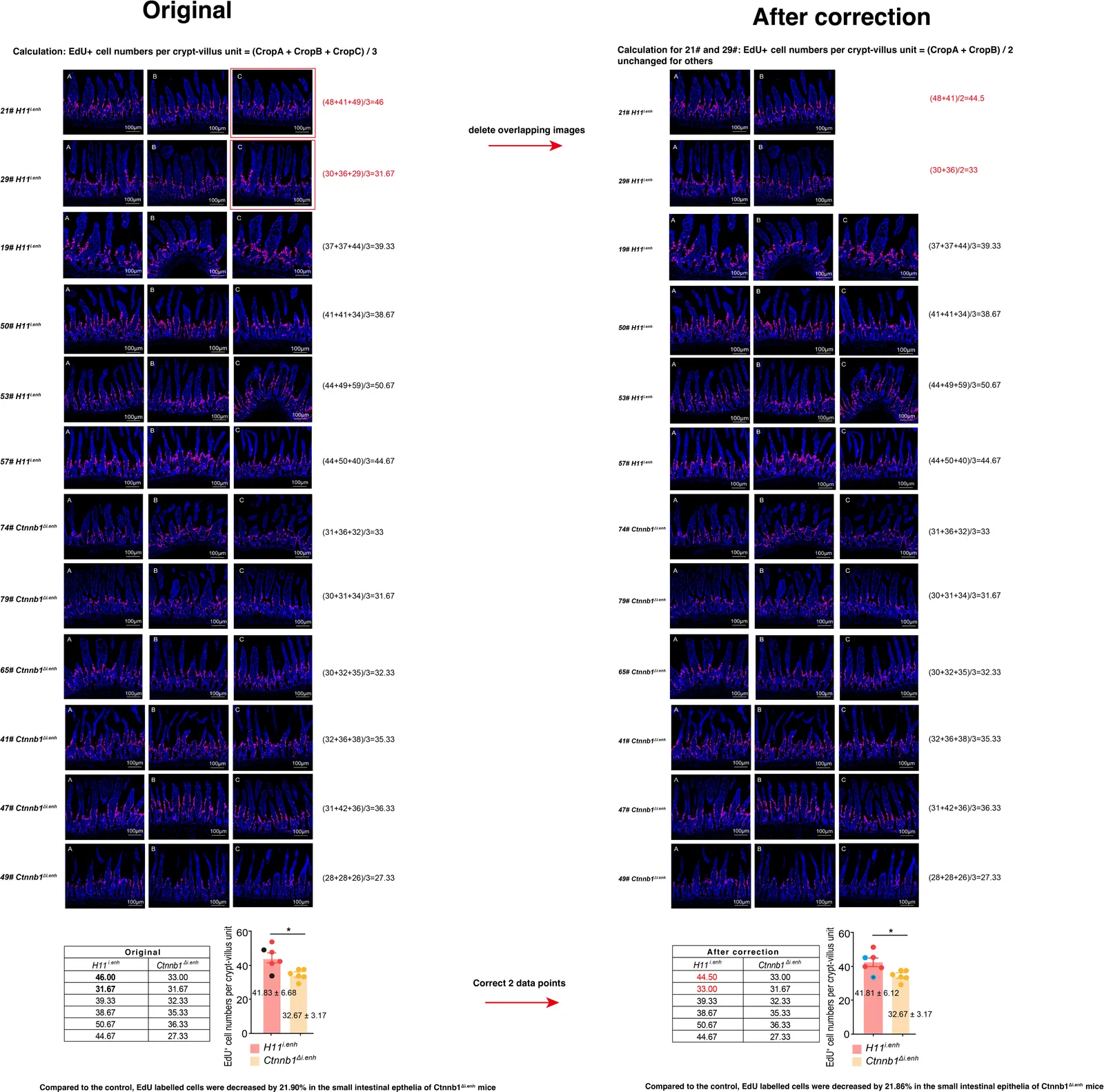


As well as correcting the chart in Figure 2, we have corrected the numerical data for Figure 2L (Figure 2—source data 6).

3. Τwo descriptive errors in the reported EdU pulse duration and the Figure 2L scale bar

The interval between EdU injection and tissue harvest should read 24 hours rather than 4 hours, and the scale bar in Figure 2L is 100 μm rather than 50 μm.

These were transcription errors introduced during manuscript preparation. The interval between EdU injection and tissue harvest was recorded as 4 hours in the main text, the Figure 2L legend and the Methods, and the scale bar for panel L was recorded as 50 μm in the Figure 2L legend. The correct values are 24 hours and 100 μm, respectively. Neither error affects the underlying images or the quantification, and both have been corrected in the main text, the Figure 2L legend and the Methods, as set out below.

We have noticed that these errors affect three locations in the article, which are corrected as follows (underline used to highlight changes):

1. Corrected Main Text:

Consistently, the numbers of Ki67-positive cells and EdU-labeled cells (24 hr pulse) were decreased by 16.1% and 21.9%, respectively, in *Ctnnb1^Δi.enh^* small intestinal epithelia (Figure 2K and L).
Original Main Text:
Consistently, the numbers of Ki67-positive cells and EdU-labeled cells (4 hr pulse) were decreased by 16.1% and 21.9%, respectively, in *Ctnnb1^Δi.enh^* small intestinal epithelia (Figure 2K and L).

2. Corrected Text in Figure 2L-N legend:

**(L)** Immunofluorescence (left and middle) and quantification (right) of EdU+ cells (red) in small intestines of *H11^i.enh^* (n=6) and *Ctnnb1^Δi.enh^* (n=6) mice after 24 hr EdU injection. Nuclei were labeled with DAPI (blue). (**M, N**) Violin plots showing expressions of marker genes for secretory cells (*Lyz1, Ang4, Muc2, Mmp7*) and absorptive cells (*Fabp6, Apoa1, Fabp2, Reg3b*) in secretory (**M**) and absorptive lineages (**N**) of *H11^i.enh^* and *Ctnnb1^Δi.enh^* small intestinal crypts. Scale bars, 50 μm (F and K), 100 μm (L).
Original Text Figure 2L-N legend:
**(L)** Immunofluorescence (left and middle) and quantification (right) of EdU+ cells (red) in small intestines of *H11^i.enh^* (n=6) and *Ctnnb1^Δi.enh^* (n=6) mice after 4 hr EdU injection. Nuclei were labeled with DAPI (blue). (**M-N**) Violin plots showing expressions of marker genes for secretory cells (*Lyz1, Ang4, Muc2, Mmp7*) and absorptive cells (*Fabp6, Apoa1, Fabp2, Reg3b*) in secretory(M) and absorptive lineages (**N**) of *H11^i.enh^* and *Ctnnb1^Δi.enh^* small intestinal crypts. Scale bars, 50 μm (F, K, and L).

3. Corrected Text in Methods:

**EdU pulse-labeling experiment**
Mice were intraperitoneally injected with EdU (10 mg/kg of body weight). After 24 hr, harvest the intestines for frozen sectioning. Staining was performed using BeyoClick EdU Cell Promotion Kit (C0075S, Beyotime, China) according to the manufacturer’s manual.
Original Text in Methods:
**EdU pulse-labeling experiment**
Mice were intraperitoneally injected with EdU (10 mg/kg of body weight). After 4 hr, harvest the intestines for frozen sectioning. Staining was performed using BeyoClick EdU Cell Promotion Kit (C0075S, Beyotime, China) according to the manufacturer’s manual.

The corrected Figure 2 (updated for panel L) is shown here:

**Figure 1:**
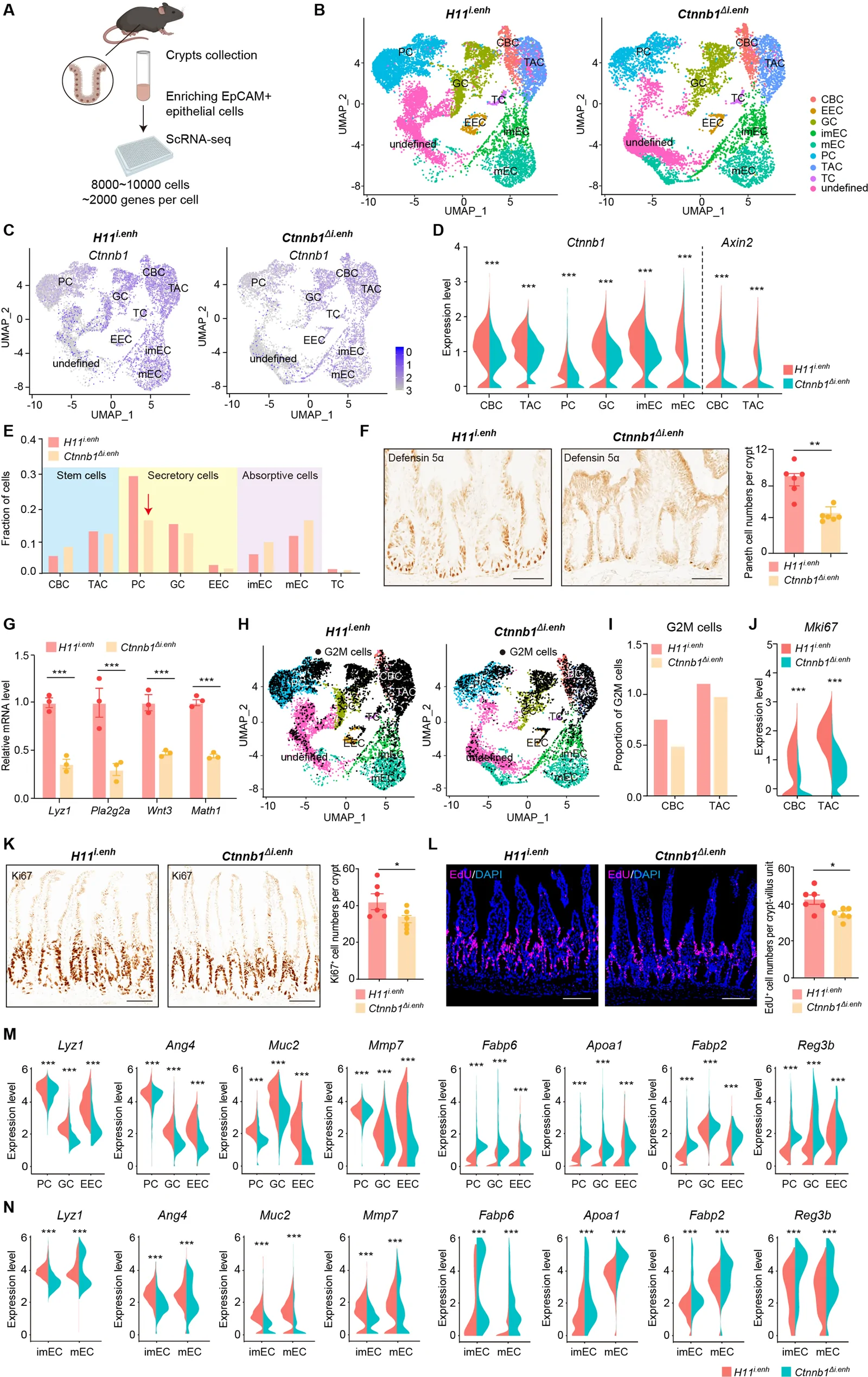


The originally published Figure 2 is shown for reference:

**Figure 2:**
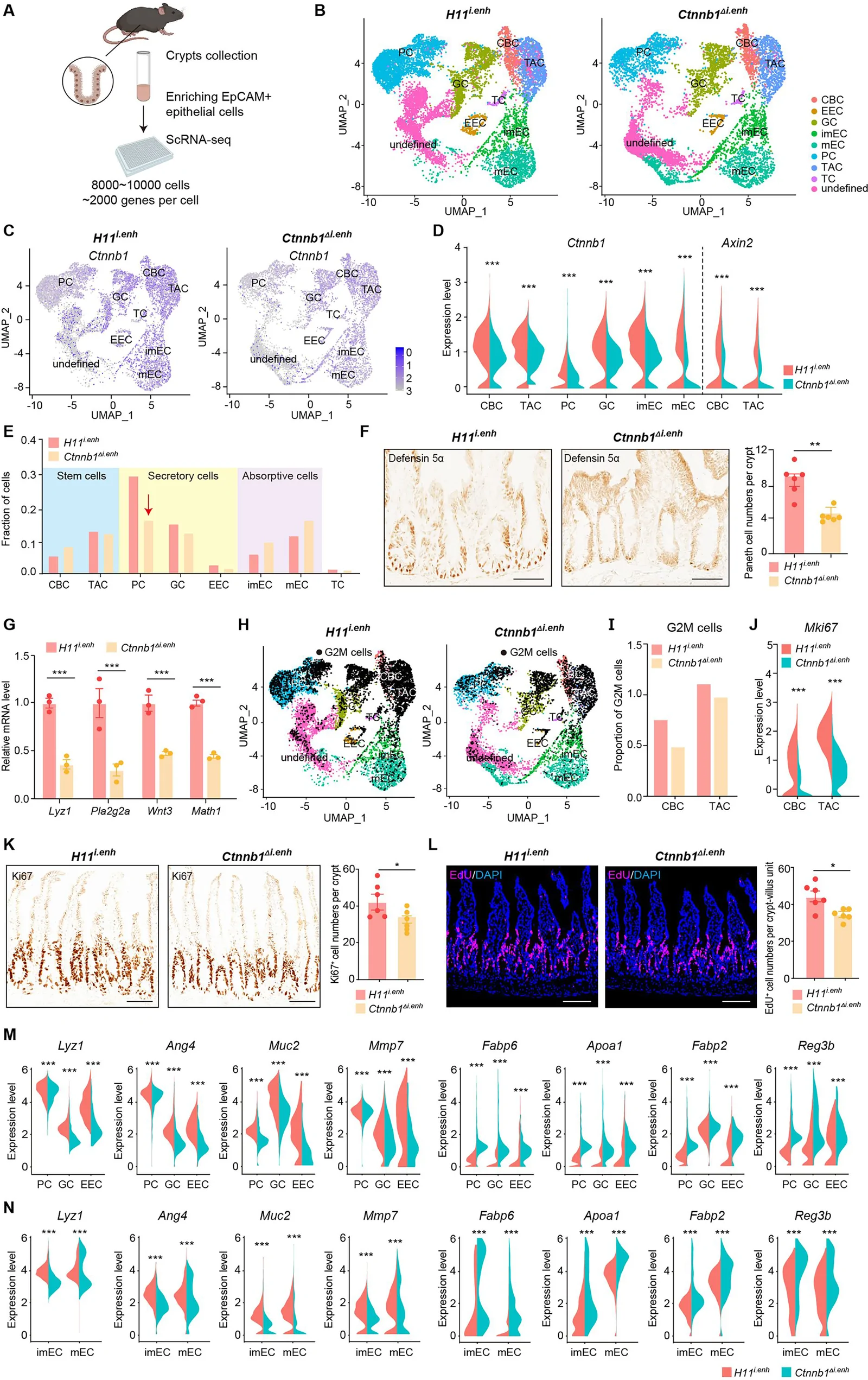


These corrections do not affect the results, interpretation or conclusions of the article. We apologize to the editors and readers for these errors.

The article has been corrected accordingly.

