# Supplementary figures and images for: A Ctnnb1 enhancer transcriptionally regulates Wnt signaling dosage to balance homeostasis and tumorigenesis of intestinal epithelia
Source: eLife. 2024 Sep 25;13:RP98238. doi: 10.7554/eLife.98238 (PMC11424096; doi:10.7554/eLife.98238)

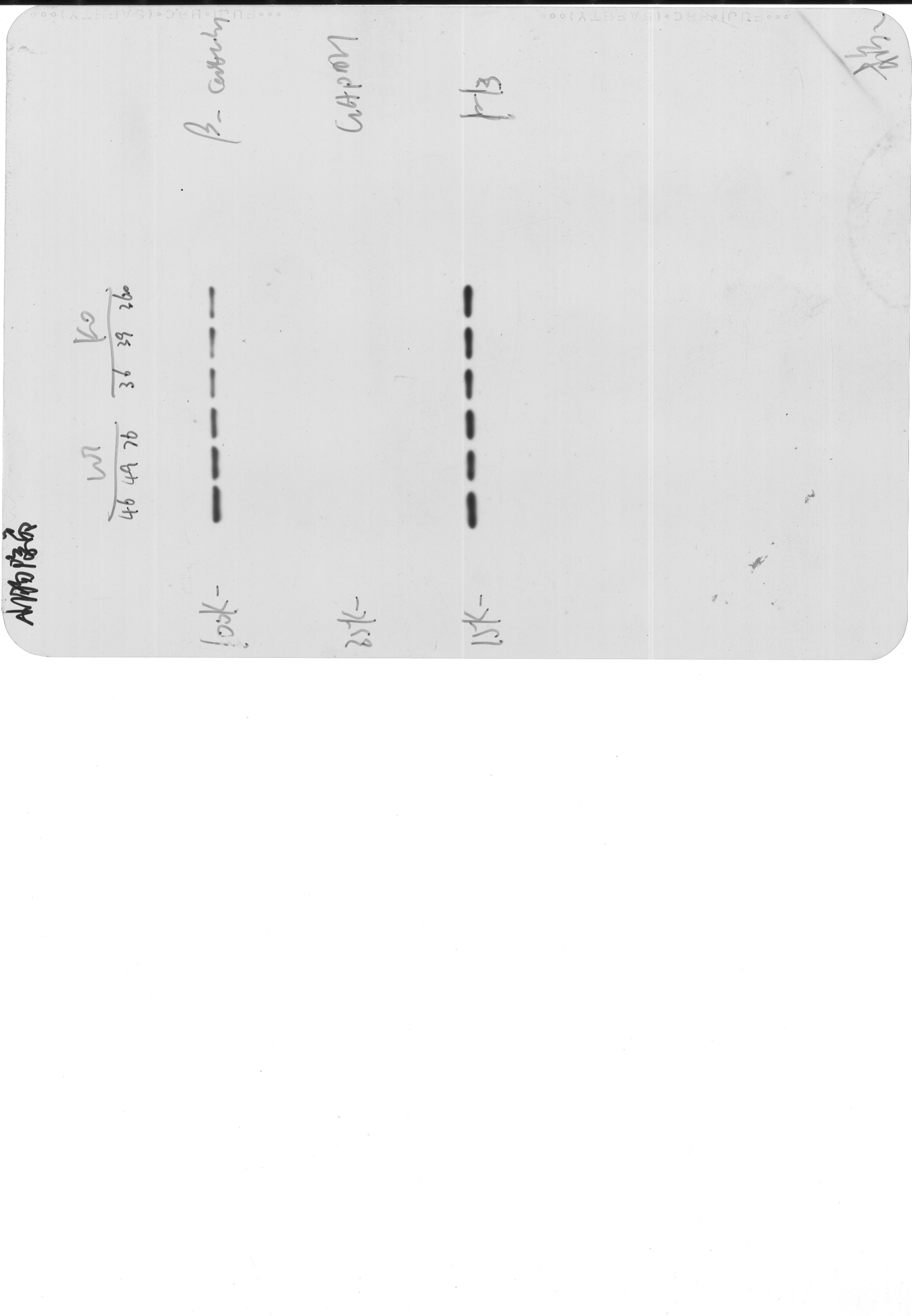

Supplement: Figure 1—source data 9. [file elife-98238-fig1-data9.zip › Figure 1-Source Data 9/Figure 1K-labelled blot.tif]

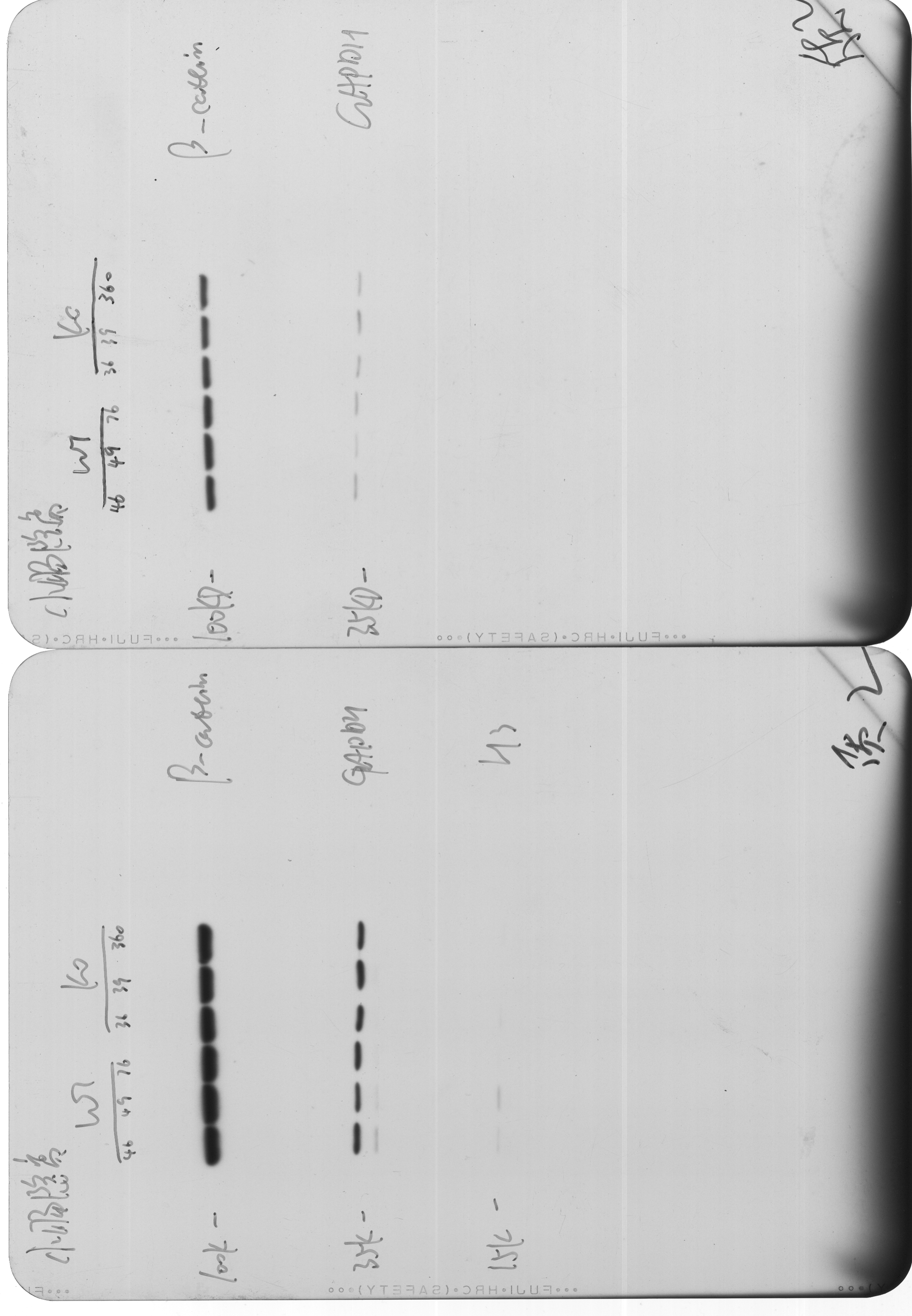

Supplement: Figure 1—source data 9. [file elife-98238-fig1-data9.zip › Figure 1-Source Data 9/Figure 1L-labelled blot.tif]

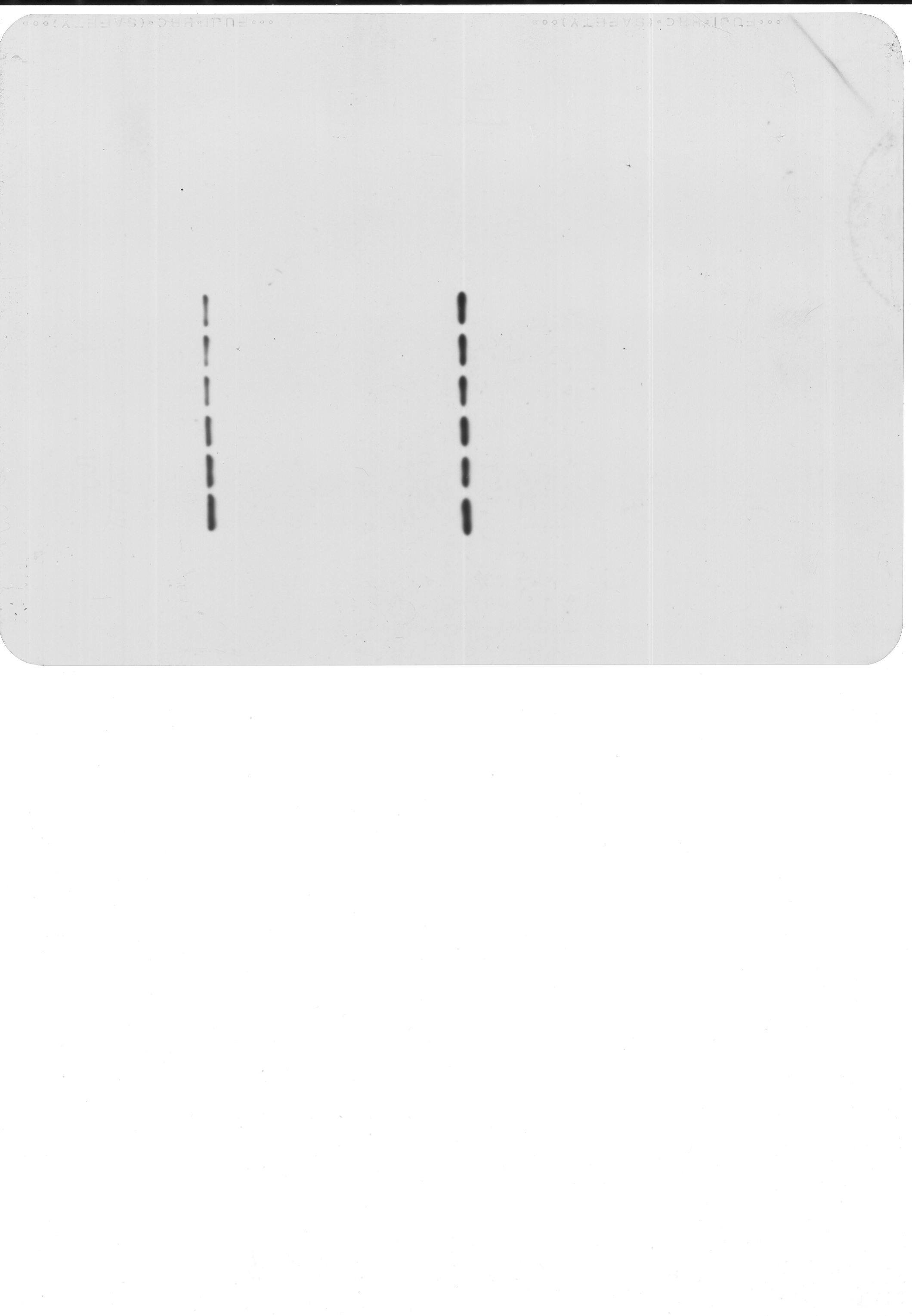

Supplement: Figure 1—source data 10. [file elife-98238-fig1-data10.zip › Figure 1-Source Data 10/Figure 1K-full raw unedited blot.tif]

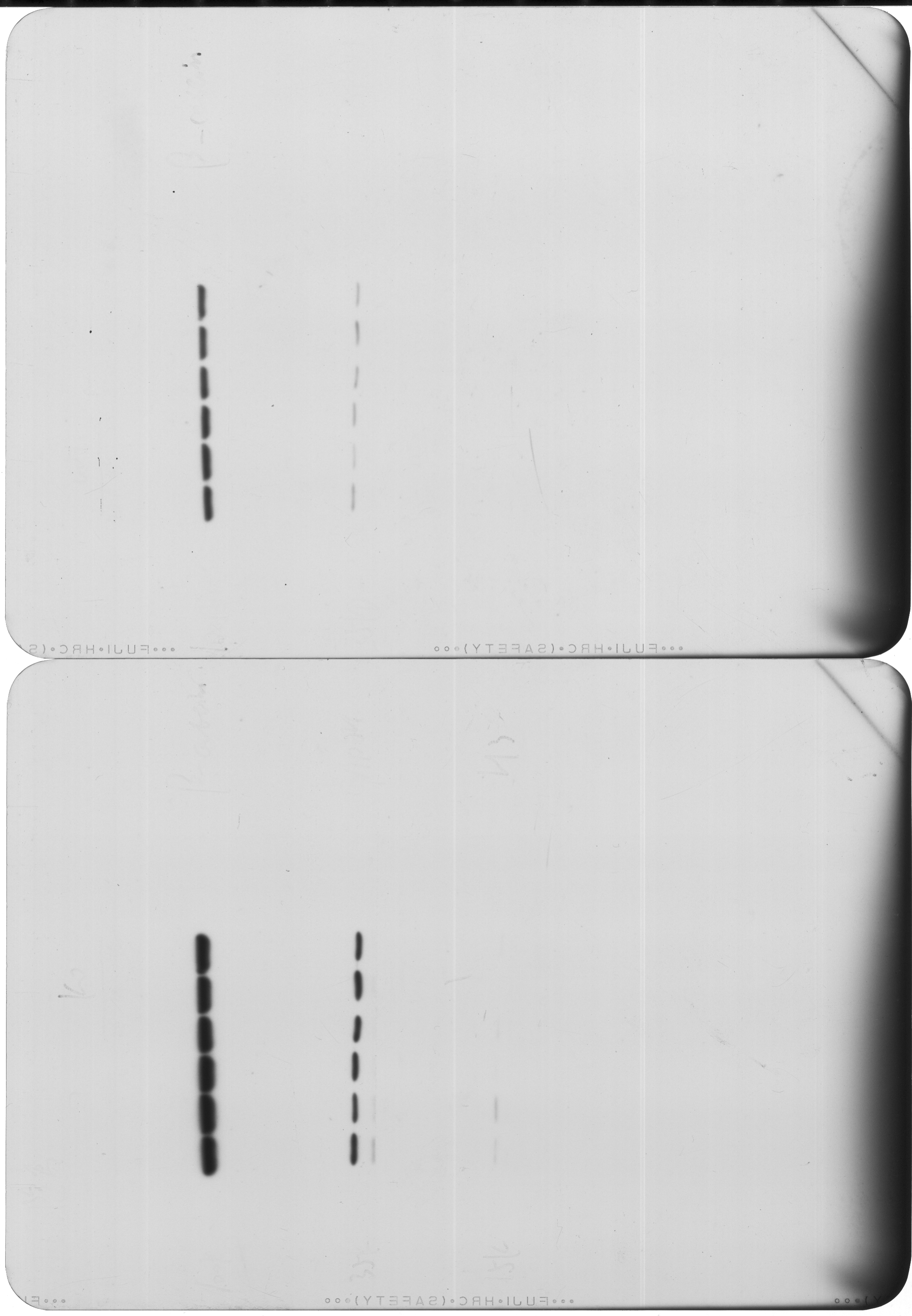

Supplement: Figure 1—source data 10. [file elife-98238-fig1-data10.zip › Figure 1-Source Data 10/Figure 1L-full raw unedited blot.tif]

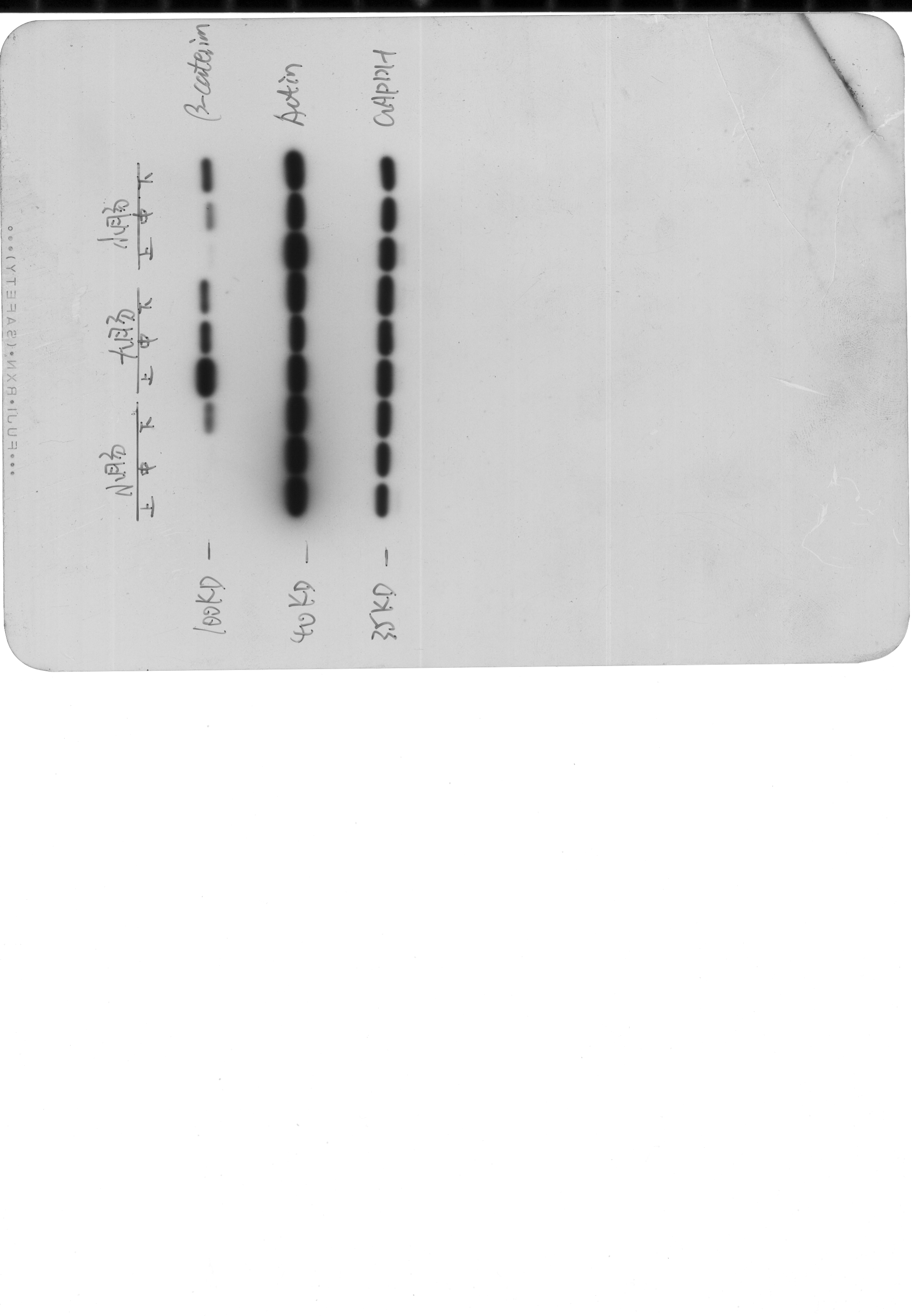

Supplement: Figure 1—figure supplement 2—source data 5. [file elife-98238-fig1-figsupp2-data5.zip › Figure 1-figure supplement 2-Source Data 5/Figure1-Figure Supplement 2E-labelled blot.tif]

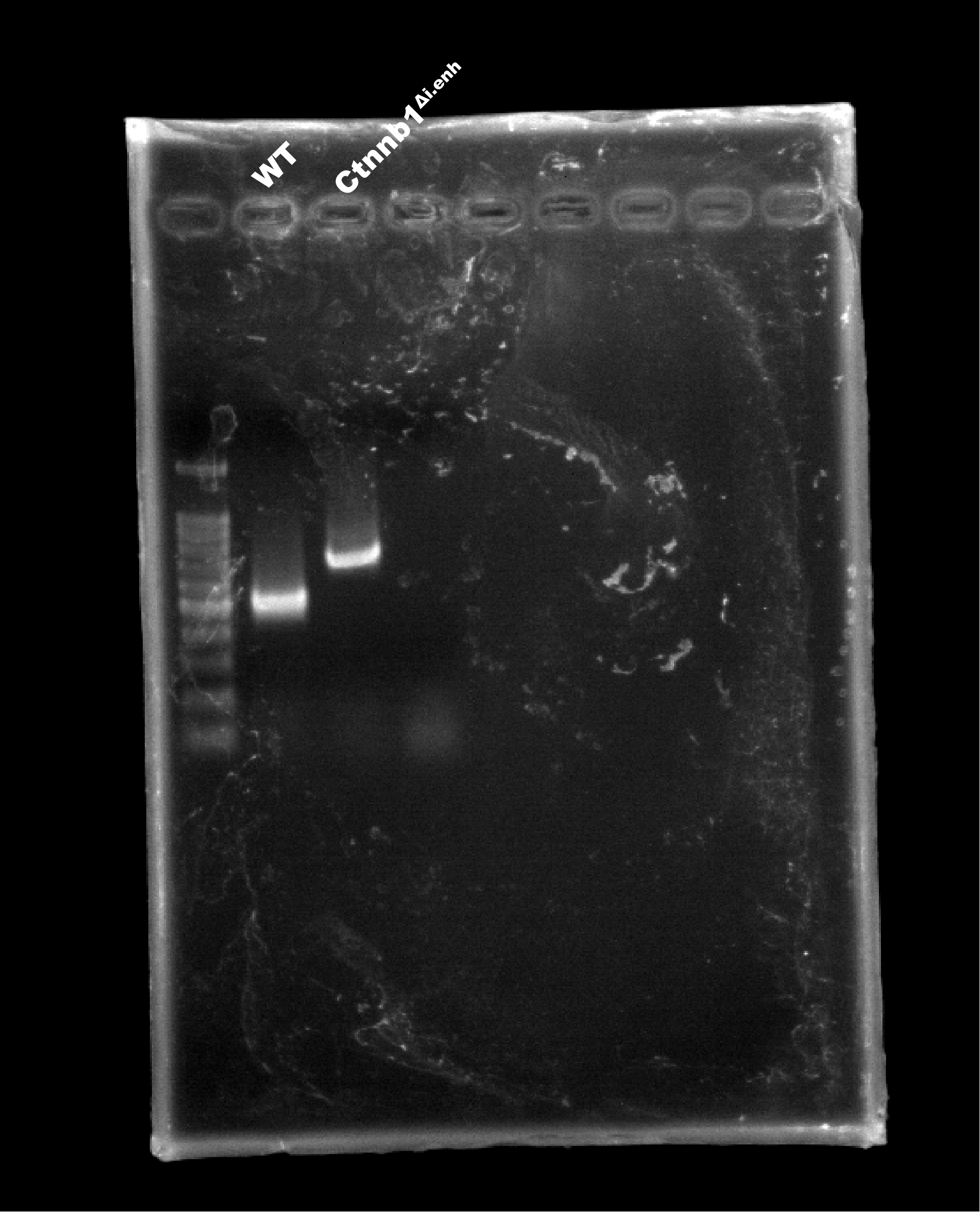

Supplement: Figure 1—figure supplement 2—source data 5. [file elife-98238-fig1-figsupp2-data5.zip › Figure 1-figure supplement 2-Source Data 5/Figure1-Figure Supplement 2I-labelled gel.jpg]

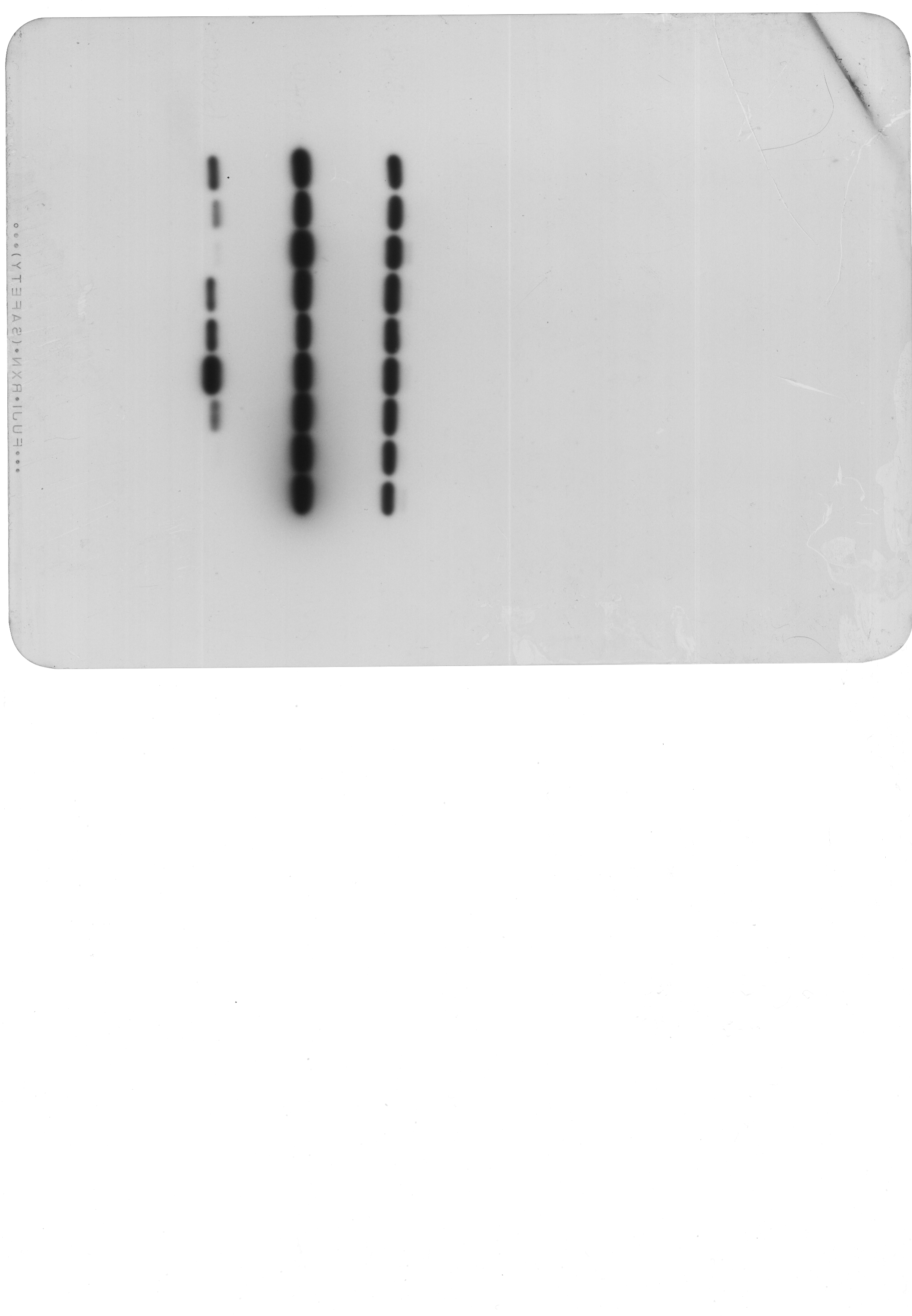

Supplement: Figure 1—figure supplement 2—source data 6. [file elife-98238-fig1-figsupp2-data6.zip › Figure 1-figure supplement 2-Source Data 6/Figure1-Figure Supplement 2E-full raw unedited blot.tif]

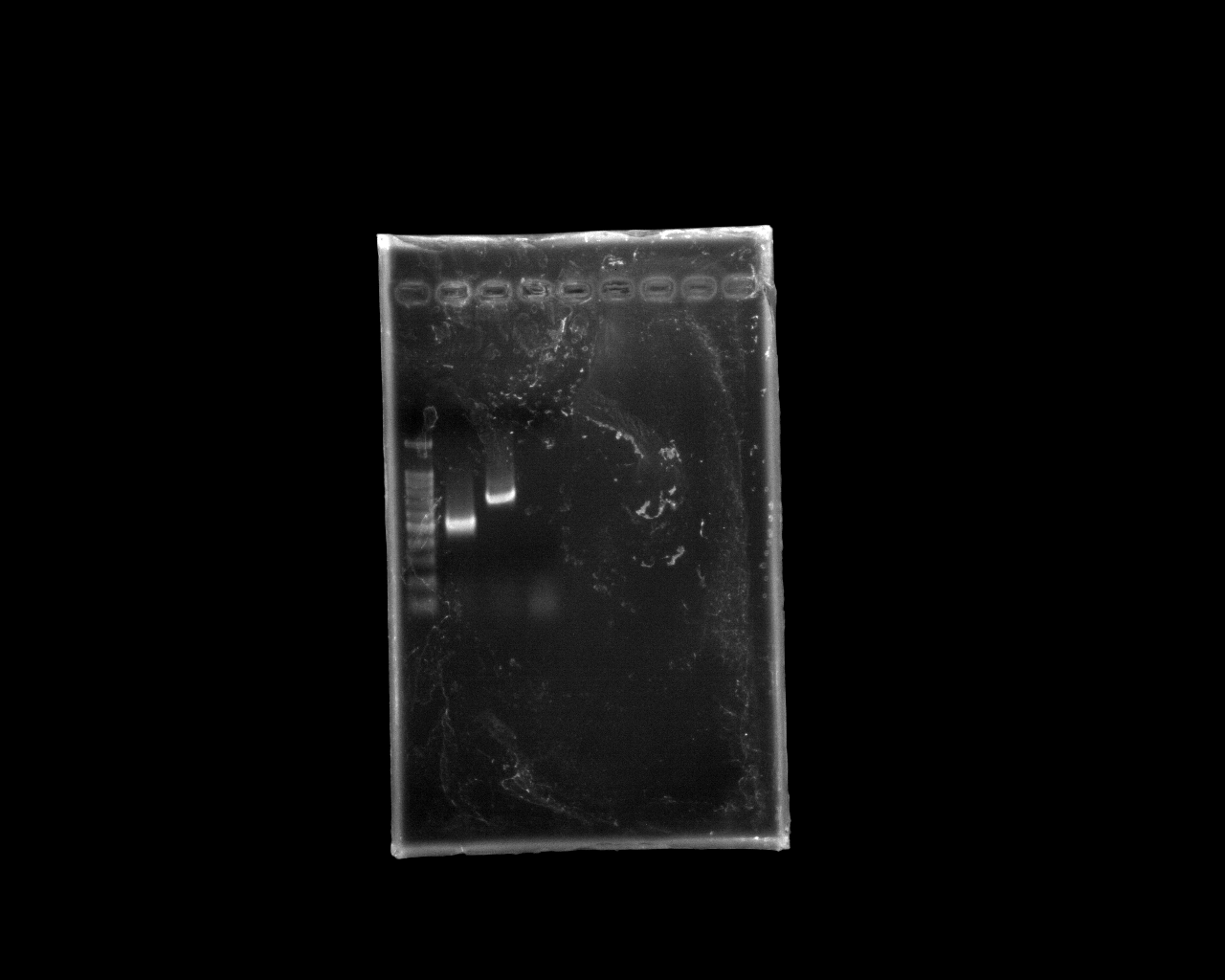

Supplement: Figure 1—figure supplement 2—source data 6. [file elife-98238-fig1-figsupp2-data6.zip › Figure 1-figure supplement 2-Source Data 6/Figure1-Figure Supplement 2I-full raw unedited gel.Tif]
