# Supplementary material for: A Ctnnb1 enhancer transcriptionally regulates Wnt signaling dosage to balance homeostasis and tumorigenesis of intestinal epithelia
Source: eLife. 2024 Sep 25;13:RP98238. doi: 10.7554/eLife.98238 (PMC11424096; doi:10.7554/eLife.98238)
Supplement: Supplementary file 1. [file elife-98238-supp1.docx]

| **Primers** | **Sequence (5'-3')** | **Description** |
| --- | --- | --- |
| shRNA-*ATOH1*-1 | cccttccagcaaacaggtgaa | used for RT-qPCR |
| shRNA-*ATOH1*-2 | agttgccggactcgcttctca | used for RT-qPCR |
| shRNA-*CDX2*-1 | caaatatcgagtggtgtacac | used for RT-qPCR |
| shRNA-*CDX2*-2 | acgtgagcatgtaccctagct | used for RT-qPCR |
| shRNA-*CREB*-1 | acggtgccaactccaatttac | used for RT-qPCR |
| shRNA-*CREB*-2 | acagcacccactagcactatt | used for RT-qPCR |
| shRNA-*PPARG*-1 | ctggcctccttgatgaataaa | used for RT-qPCR |
| shRNA-*PPARG*-2 | gacaacagacaaatcaccatt | used for RT-qPCR |
| shRNA-*VDR*-1 | ttggctttgctaagatgatac | used for RT-qPCR |
| shRNA-*VDR*-2 | cctccagttcgtgtgaatgat | used for RT-qPCR |
| shRNA-*HNF4A*-1 | tcacctgatgcaggaacatat | used for RT-qPCR |
| shRNA-*HNF4A*-2 | tcttcggcatggccaagattg | used for RT-qPCR |
| shRNA-*HNF4G*-1 | gtgacagaataagcaccagaa | used for RT-qPCR |
| shRNA-*HNF4G*-2 | cgtggcaaatgattgagcaaa | used for RT-qPCR |
| shRNA-*KDM6A*-1 | ccgcgcaaatagaaataattt | used for RT-qPCR |
| shRNA-*KDM6A*-2 | gcagcacgaattaagtattta | used for RT-qPCR |
| *ATOH1*-F | ccttccagcaaacaggtgaat | used for RT-qPCR |
| *ATOH1*-R | ttgttgaacgacgggataaca | used for RT-qPCR |
| *CREB*-F | attcacaggagtcagtggatagt | used for RT-qPCR |
| *CREB*-R | caccgttacagtggtgatgg | used for RT-qPCR |
| *PPARG*-F | gatgccagcgactttgactc | used for RT-qPCR |
| *PPARG*-R | acccacgtcatcttcaggga | used for RT-qPCR |
| *CDX2*-F | gacgtgagcatgtaccctagc | used for RT-qPCR |
| *CDX2-*R | gcgtagccattccagtcct | used for RT-qPCR |
| *VDR*-F | gtggacatcggcatgatgaag | used for RT-qPCR |
| *VDR*-R | ggtcgtaggtcttatggtggg | used for RT-qPCR |
| *HNF4A*-F | cacgggcaaacactacggt | used for RT-qPCR |
| *HNF4A*-R | ttgaccttcgagtgctgatcc | used for RT-qPCR |
| *HNF4G*-F | ttgcaggttcagtcggcaat | used for RT-qPCR |
| *HNF4G*-R | tttcattcccgctctaaaacact | used for RT-qPCR |
| *KDM6A*-F | ttcctcggaaggtgctattca | used for RT-qPCR |
| *KDM6A*-R | gaggctggttgcaggattca | used for RT-qPCR |
| *Ctnnb1*-F | atggagccggacagaaaagc | used for RT-qPCR |
| *Ctnnb1*-R | cttgccactcagggaagga | used for RT-qPCR |
| *CTNNB1*-F | aaagcggctgttagtcactgg | used for RT-qPCR |
| *CTNNB1*-R | cgagtcattgcatactgtccat | used for RT-qPCR |
| *Gapdh*-F | aggtcggtgtgaacggatttg | used for RT-qPCR |
| *Gapdh*-R | tgtagaccatgtagttgaggtca | used for RT-qPCR |
| *ACTB*-F | agtgcgacgtggacatccg | used for RT-qPCR |
| *ACTB*-R | tggctctaacagtccgcctag | used for RT-qPCR |
| ieCtnnb1-ChIP-1F | tgactaaggacaggcctttcccc | used for ChIP-qPCR |
| ieCtnnb1-ChIP-1R | ggcagtcacaggtcaggtctcat | used for ChIP-qPCR |
| ieCtnnb1-ChIP-2F | gcagaggtaccagagcaaaggtg | used for ChIP-qPCR |
| ieCtnnb1-ChIP-2R | gctggaatccttgaagacctgct | used for ChIP-qPCR |
| ieCtnnb1-ChIP-3F | ctgacgtcttgacattcctgttga | used for ChIP-qPCR |
| ieCtnnb1-ChIP-3R | gcctgaacgatggagattacatcc | used for ChIP-qPCR |
| ieCtnnb1-ChIP-4F | cctcctgagagttctgccatcct | used for ChIP-qPCR |
| ieCtnnb1-ChIP-4R | gcttgaacaggcagactttgtaacc | used for ChIP-qPCR |
| ieCtnnb1-ChIP-5F | tttgtggcaagtcttggacagct | used for ChIP-qPCR |
| ieCtnnb1-ChIP-5R | tggttctgccttgtgatcagcaa | used for ChIP-qPCR |
| ieCtnnb1-ChIP-6F | ctgtcagagggcaaagccgttta | used for ChIP-qPCR |
| ieCtnnb1-ChIP-6R | tgtgggaatcgggaagctctg | used for ChIP-qPCR |
| ieCtnnb1-ChIP-7F | gcttctttccgggggcaaagt | used for ChIP-qPCR |
| ieCtnnb1-ChIP-7R | gggaatgtcaggcacagtgca | used for ChIP-qPCR |
| *pCtnnb1*-ChIP-1F | gcagatgtctcagtgcagtctct | used for ChIP-qPCR |
| *pCtnnb1*-ChIP-1R | ccgaaatacaaaggccacaagct | used for ChIP-qPCR |
| *pCtnnb1*-ChIP-2F | gcttgtggcctttgtatttcggt | used for ChIP-qPCR |
| *pCtnnb1*-ChIP-2R | cggggatttctgaatgtttaaggtga | used for ChIP-qPCR |
| *pCtnnb1*-ChIP-3F | ccccgaaattaaaatgaagtgcctc | used for ChIP-qPCR |
| *pCtnnb1*-ChIP-3R | ggacaaaggttaggtaggtcacaga | used for ChIP-qPCR |
| *pCtnnb1*-ChIP-4F | acctttgtcctcaaggccgag | used for ChIP-qPCR |
| *pCtnnb1*-ChIP-4R | gcctgggagggtttgttgtagag | used for ChIP-qPCR |
| *pCtnnb1*-ChIP-5F | caaaccctcccaggctgaagtc | used for ChIP-qPCR |
| *pCtnnb1*-ChIP-5R | ttacagactgtgaggcccagga | used for ChIP-qPCR |
| *pCtnnb1*-ChIP-6F | gggaaacatttaatcgaatgcaggcg | used for ChIP-qPCR |
| *pCtnnb1*-ChIP-6R | tcaggaagactgactgagaagcac | used for ChIP-qPCR |
| *pCtnnb1*-ChIP-7F | caactgttatggtgaccccaacc | used for ChIP-qPCR |
| *pCtnnb1*-ChIP-7R | ggtcacctaagaccgtgggaaaa | used for ChIP-qPCR |
| ieCTNNB1-ChIP-1F | tttctagggcatctgctgcaaag | used for ChIP-qPCR |
| ieCTNNB1-ChIP-1R | gacaaactataagtggccattgacca | used for ChIP-qPCR |
| ieCTNNB1-ChIP-2F | cacagacagttctattaaacaacact | used for ChIP-qPCR |
| ieCTNNB1-ChIP-2R | gtgagagtggaaggtggattaaaaa | used for ChIP-qPCR |
| ieCTNNB1-ChIP-3F | ggggatgttgtaggtgctcaata | used for ChIP-qPCR |
| ieCTNNB1-ChIP-3R | gaataaatcaggcccacttccaa | used for ChIP-qPCR |
| ieCTNNB1-ChIP-4F | tttggtagctgattgagagcttg | used for ChIP-qPCR |
| ieCTNNB1-ChIP-4R | gctcccataaattatgtcaccattc | used for ChIP-qPCR |
| ieCTNNB1-ChIP-5F | gtcatacctacccattacctcttc | used for ChIP-qPCR |
| ieCTNNB1-ChIP-5R | taaagagacacttgtcctcatgg | used for ChIP-qPCR |
| ieCTNNB1-ChIP-6F | tcctggagttcctgtaacccagtt | used for ChIP-qPCR |
| ieCTNNB1-ChIP-6R | tactgcttccaactggctgtgtg | used for ChIP-qPCR |
| *pCTNNB1*-ChIP-1F | gctgcttaatcgatagctttctc | used for ChIP-qPCR |
| *pCTNNB1*-ChIP-1R | ccacttgtcactaggtatcaatag | used for ChIP-qPCR |
| *pCTNNB1*-ChIP-2F | gcgctctggagctaatccatttc | used for ChIP-qPCR |
| *pCTNNB1*-ChIP-2R | aaggctgtgaactctccgtagaa | used for ChIP-qPCR |
| *pCTNNB1*-ChIP-3F | gctgaacagcctgctgagaggt | used for ChIP-qPCR |
| *pCTNNB1*-ChIP-3R | ttgtggtctgtgccgcacactc | used for ChIP-qPCR |
| *pCTNNB1*-ChIP-4F | gagatgccaccttccgcagg | used for ChIP-qPCR |
| *pCTNNB1*-ChIP-4R | aaggtggccctggtatcctc | used for ChIP-qPCR |
| *pCTNNB1*-ChIP-5F | atgcagaccacagcgccctca | used for ChIP-qPCR |
| *pCTNNB1*-ChIP-5R | agcagtctgctgccgtctgag | used for ChIP-qPCR |
| *pCTNNB1*-ChIP-6F | ggtctgaggagcagcttcagtc | used for ChIP-qPCR |
| *pCTNNB1*-ChIP-6R | ataaggaaaggagcgcccaagc | used for ChIP-qPCR |
| sg1 | tgactccaactacaagcgag | used for CRISPRa andCRISPRi |
| sg2 | ttacgagctgccaactctca | used for CRISPRa andCRISPRi |
| sg3 | ctgtctaggtaggcggtagt | used for CRISPRa andCRISPRi |
| *Ctnnb1^Δi.enh^* -WT-F | gtcctgtccgtcactattatcctggc | used for validation of  *Ctnnb1^Δi.enh^* mice |
| *Ctnnb1^Δi.enh^* -WT-R | ccactgccctgctaaagcattggt | used for validation of  *Ctnnb1^Δi.enh^* mice |
| *Ctnnb1^Δi.enh^* -Mut-R | tgattagttctccgggaagcccagt | used for validation of  *Ctnnb1^Δi.enh^* mice |
| *APC^Min/+^*-F | atctcatggcaaacagacct | used for validation of *APC^Min/+^*mice |
| *APC^Min/+^* -R | tcacaaatcatctcgcaga | used for validation of *APC^Min/+^*mice |
| *Lgr5-EGFP*-WT-F | ctgctctctgctcccagtct | used for validation of  Lgr5-EGFP mice |
| *Lgr5-EGFP*-WT-R | ataccccatcccttttgagc | used for validation of  Lgr5-EGFP mice |
| *Lgr5-EGFP*-Mut-R | gaacttcagggtcagcttgc | used for validation of  Lgr5-EGFP mice |
| *LacZ* F | atcctctgcatggtcaggtc | used for validation of *H11^i.enh^/ H11^hi.enh^* and BAT-Gal mice |
| *LacZ* R | cgtggcctgattcattcc | used for validation of *H11^i.enh^/ H11^hi.enh^* and BAT-Gal mice |
| sgRNA-1 | tgttggctcagcagacacccagg | used for construction of *H11^i.enh^* mice |
| sgRNA-2 | actgcctcctcagcttcaagagg | used for construction of *H11^i.enh^* mice |
| EGE-WL-020-T7-sgRNA3 | ctatttctagctctaaaactttgggg atcaagtaaggggcctatagtgagt cgtatta | used for construction of *Ctnnb1^Δi.enh^* mice |
| EGE-WL-020-T7-sgRNA13 | ctatttctagctctaaaaccctcatca  taggaaggcttcctatagtgagtcgt  atta | used for construction of *Ctnnb1^Δi.enh^* mice |
