# Supplementary material for: A Ctnnb1 enhancer transcriptionally regulates Wnt signaling dosage to balance homeostasis and tumorigenesis of intestinal epithelia
Source: eLife. 2024 Sep 25;13:RP98238. doi: 10.7554/eLife.98238 (PMC11424096; doi:10.7554/eLife.98238)
Supplement: Supplementary file 2. [file elife-98238-supp2.docx]

| **Tissue** | **Species** | **Age** | **Strategy** | **Antibody** | **Serial number** |
| --- | --- | --- | --- | --- | --- |
| Intestine | Mus musculus | E14.5 | ChIP-seq | H3K27ac | ENCSR424END |
| Intestine | Mus musculus | E15.5 | ChIP-seq | H3K27ac | ENCSR599GVS |
| Intestine | Mus musculus | E16.5 | ChIP-seq | H3K27ac | ENCSR639DND |
| Intestine | Mus musculus | P0 | ChIP-seq | H3K27ac | ENCSR642VYW |
| Small Intestine | Mus musculus | 2 months | ChIP-seq | H3K27ac | ENCSR000CCQ |
| Intestine | Mus musculus | E14.5 | ChIP-seq | H3K4me3 | ENCSR464MQU |
| Intestine | Mus musculus | E15.5 | ChIP-seq | H3K4me3 | ENCSR410YIY |
| Intestine | Mus musculus | E16.5 | ChIP-seq | H3K4me3 | ENCSR572KYR |
| Intestine | Mus musculus | P0 | ChIP-seq | H3K4me3 | ENCSR198ACZ |
| Small Intestine | Mus musculus | 2 months | ChIP-seq | H3K4me3 | ENCSR000CCS |
| Intestine | Mus musculus | E14.5 | ChIP-seq | H3K4me1 | ENCSR157LYR |
| Intestine | Mus musculus | E15.5 | ChIP-seq | H3K4me1 | ENCSR051CUH |
| Intestine | Mus musculus | E16.5 | ChIP-seq | H3K4me1 | ENCSR829YGD |
| Intestine | Mus musculus | P0 | ChIP-seq | H3K4me1 | ENCSR159RVN |
| Small Intestine | Mus musculus | 2 months | ChIP-seq | H3K4me1 | ENCSR000CCR |
| Intestine | Mus musculus | E14.5 | DNase-seq | / | ENCSR655WKX |
| Large Intestine | Mus musculus | 2 months | DNase-seq | / | ENCSR000CNH |
| Intestine | Mus musculus | E14.5 | ChIP-seq | H3k36me3 | ENCSR953KTY |
| Intestine | Mus musculus | E15.5 | ChIP-seq | H3k36me3 | ENCSR919DDC |
| Intestine | Mus musculus | E16.5 | ChIP-seq | H3k36me3 | ENCSR272XPJ |
| Intestine | Mus musculus | P0 | ChIP-seq | H3k36me3 | ENCSR483KOD |
| Small Intestine | Mus musculus | 2 months | ChIP-seq | H3k36me3 | ENCSR000CFS |
| Stomach | Mus musculus | E14.5 | ChIP-seq | H3K27ac | ENCSR316CNR |
| Stomach | Mus musculus | E15.5 | ChIP-seq | H3K27ac | ENCSR929SEW |
| Stomach | Mus musculus | E16.5 | ChIP-seq | H3K27ac | ENCSR546ANT |
| Stomach | Mus musculus | P0 | ChIP-seq | H3K27ac | ENCSR346FJG |
| Stomach | Mus musculus | E15.5 | ChIP-seq | H3K4me1 | ENCSR548BKP |
| Stomach | Mus musculus | E14.5 | ChIP-seq | H3K4me1 | ENCSR335WME |
| Stomach | Mus musculus | E16.5 | ChIP-seq | H3K4me1 | ENCSR907CPZ |
| Stomach | Mus musculus | P0 | ChIP-seq | H3K4me1 | ENCSR940CMI |
| Stomach | Mus musculus | E15.5 | ChIP-seq | H3K4me3 | ENCSR522LXN |
| Stomach | Mus musculus | E14.5 | ChIP-seq | H3K4me3 | ENCSR023VJO |
| Stomach | Mus musculus | E16.5 | ChIP-seq | H3K4me3 | ENCSR684UWM |
| Stomach | Mus musculus | P0 | ChIP-seq | H3K4me3 | ENCSR916CBN |
| Stomach | Mus musculus | P0 | DNase-seq | / | ENCSR969OPE |
| Stomach | Mus musculus | E14.5 | ChIP-seq | H3k36me3 | ENCSR581FAT |
| Stomach | Mus musculus | E15.5 | ChIP-seq | H3k36me3 | ENCSR599PKR |
| Stomach | Mus musculus | E16.5 | ChIP-seq | H3k36me3 | ENCSR872WGX |
| Stomach | Mus musculus | P0 | ChIP-seq | H3k36me3 | ENCSR516KLO |
| Liver | Mus musculus | E14.5 | ChIP-seq | H3K27ac | ENCSR075SNV |
| Liver | Mus musculus | E15.5 | ChIP-seq | H3K27ac | ENCSR479LFP |

| Liver | Mus musculus | E16.5 | ChIP-seq | H3K27ac | ENCSR802RET |
| --- | --- | --- | --- | --- | --- |
| Liver | Mus musculus | P0 | ChIP-seq | H3K27ac | ENCSR616TJM |
| Liver | Mus musculus | 2 months | ChIP-seq | H3K27ac | ENCSR000CDH |
| Liver | Mus musculus | E14.5 | ChIP-seq | H3K4me3 | ENCSR433ESG |
| Liver | Mus musculus | E15.5 | ChIP-seq | H3K4me3 | ENCSR577SDJ |
| Liver | Mus musculus | E16.5 | ChIP-seq | H3K4me3 | ENCSR252GKD |
| Liver | Mus musculus | P0 | ChIP-seq | H3K4me3 | ENCSR653AVN |
| Liver | Mus musculus | 2 months | ChIP-seq | H3K4me3 | ENCSR000CAP |
| Liver | Mus musculus | E14.5 | ChIP-seq | H3K4me1 | ENCSR000CDW |
| Liver | Mus musculus | E15.5 | ChIP-seq | H3K4me1 | ENCSR133EGP |
| Liver | Mus musculus | E16.5 | ChIP-seq | H3K4me1 | ENCSR487OLC |
| Liver | Mus musculus | P0 | ChIP-seq | H3K4me1 | ENCSR308GFM |
| Liver | Mus musculus | 2 months | ChIP-seq | H3K4me1 | ENCSR000CAO |
| Liver | Mus musculus | E14.5 | DNase-seq | / | ENCSR000CNJ |
| Liver | Mus musculus | E14.5 | ChIP-seq | H3k36me3 | ENCSR670YXP |
| Liver | Mus musculus | E15.5 | ChIP-seq | H3k36me4 | ENCSR510CGB |
| Liver | Mus musculus | E16.5 | ChIP-seq | H3k36me5 | ENCSR569DBO |
| Liver | Mus musculus | P0 | ChIP-seq | H3k36me6 | ENCSR656AMS |
| Liver | Mus musculus | 2 months | ChIP-seq | H3k36me7 | ENCSR000CEO |
| Forebrain | Mus musculus | P0 | ChIP-seq | H3K27ac | ENCSR094TTT |
| Heart | Mus musculus | 2 months | ChIP-seq | H3K27ac | ENCSR000CDF |
| Lung | Mus musculus | P0 | ChIP-seq | H3K27ac | ENCSR884MYD |
| Limb | Mus musculus | E15.5 | ChIP-seq | H3K27ac | ENCSR988BRP |
| Craniofacial | Mus musculus | E14.5 | ChIP-seq | H3K27ac | ENCSR481SGM |
| Cortical plate | Mus musculus | 2 months | ChIP-seq | H3K27ac | ENCSR000CDD |
| Midbrain | Mus musculus | P0 | ChIP-seq | H3K27ac | ENCSR672ZXY |
| Hindbrain | Mus musculus | P0 | ChIP-seq | H3K27ac | ENCSR332JYZ |
| Testis | Mus musculus | 2 months | ChIP-seq | H3K27ac | ENCSR000CCU |
| Cerebellum | Mus musculus | 2 months | ChIP-seq | H3K27ac | ENCSR000CDC |
| Gastrocnemius | Mus musculus | 2 months | ChIP-seq | H3K27ac | ENCSR714ZJT |
| Kidney | Mus musculus | 2 months | ChIP-seq | H3K27ac | ENCSR000CDG |
| Small Intestine | Homo sapiens | 30 years | ChIP-seq | H3K27ac | ENCSR655XLM |
| Sigmoid colon | Homo sapiens | 34 years | ChIP-seq | H3K27ac | ENCSR561YSH |
| Small Intestine | Homo sapiens | 30 years | ChIP-seq | H3K4me3 | ENCSR944QSH |
| Sigmoid colon | Homo sapiens | 34 years | ChIP-seq | H3K4me3 | ENCSR792IJA |
| Small Intestine | Homo sapiens | 30 years | ChIP-seq | H3K4me1 | ENCSR538JMW |
| Sigmoid colon | Homo sapiens | 34 years | ChIP-seq | H3K4me1 | ENCSR782OZZ |
| Small Intestine | Homo sapiens | 30 years | ChIP-seq | H3K36me3 | ENCSR073YZL |
| Sigmoid colon | Homo sapiens | 34 years | ChIP-seq | H3K36me3 | ENCSR445RFF |
| Small Intestine | Homo sapiens | 34 years | DNase-seq | / | ENCSR931UQB |
| Sigmoid colon | Homo sapiens | 37 years | DNase-seq | / | ENCSR923JYH |
| Large intestine | Mus musculus | 2 months | DNase-seq | / | ENCSR000CNH |
| Hippocampus | Homo sapiens | 73 years | ChIP-seq | H3K27ac | ENCSR321LKT |
| Kidney | Homo sapiens | 50 years | ChIP-seq | H3K27ac | ENCSR438SPO |
| Urinary bladder | Homo sapiens | 34 years | ChIP-seq | H3K27ac | ENCSR054BKO |
| Muscle of leg | Homo sapiens | 110 days | ChIP-seq | H3K27ac | ENCSR687ZCM |
| Cingulate gyrus | Homo sapiens | 75 years | ChIP-seq | H3K27ac | ENCSR604JDV |
| Spleen | Homo sapiens | 30 years | ChIP-seq | H3K27ac | ENCSR086XCT |
| Ovary | Homo sapiens | 30 years | ChIP-seq | H3K27ac | ENCSR268JQE |
| Testis | Homo sapiens | 37 years | ChIP-seq | H3K27ac | ENCSR136ZQZ |
| Adrenal gland | Homo sapiens | 30 years | ChIP-seq | H3K27ac | ENCSR642HHF |
| Esophagus | Homo sapiens | 30 years | ChIP-seq | H3K27ac | ENCSR645SYH |
| Small Intestine | Mus musculus | Adult | ChIP-seq | HNF4𝛼 | GSM851120 |
| Organoids | Mus musculus | / | ChIP-seq | HNF4G | GSM3132969 |
| Colon | Mus musculus | Adult | ChIP-seq | ATOH1 | [GSM2185705](http://www.ncbi.nlm.nih.gov/geo/query/acc.cgi?acc=GSM2185705) |
| Intestinal villus | Mus musculus | Adult | ChIP-seq | KDM6A | [GSM2610642](http://www.ncbi.nlm.nih.gov/geo/query/acc.cgi?acc=GSM2610642) |
| Intestinal villus | Mus musculus | Adult | ChIP-seq | CDX2 | [GSM2610627](http://www.ncbi.nlm.nih.gov/geo/query/acc.cgi?acc=GSM2610627) |
| Duodenum | Mus musculus | Adult | ChIP-seq | VDR | [GSM1694861](http://www.ncbi.nlm.nih.gov/geo/query/acc.cgi?acc=GSM1694861) |
| Caco-2 | Homo sapiens | / | ChIP-seq | HNF4𝛼 | GSM575229 |
| LS180 | Homo sapiens | / | ChIP-seq | CDX2 | [GSM791413](http://www.ncbi.nlm.nih.gov/geo/query/acc.cgi?acc=GSM791413) |
| HT29 | Homo sapiens | / | ChIP-seq | PPARG | [GSM2042856](http://www.ncbi.nlm.nih.gov/geo/query/acc.cgi?acc=GSM2042856) |
| LoVo | Homo sapiens | / | ChIP-seq | CREB1 | [GSM1239450](http://www.ncbi.nlm.nih.gov/geo/query/acc.cgi?acc=GSM1239450) |
