# Supplementary material for: A Ctnnb1 enhancer transcriptionally regulates Wnt signaling dosage to balance homeostasis and tumorigenesis of intestinal epithelia
Source: eLife. 2024 Sep 25;13:RP98238. doi: 10.7554/eLife.98238 (PMC11424096; doi:10.7554/eLife.98238)
Supplement: Supplementary file 3. [file elife-98238-supp3.docx]

|  | Name | Matrix ID | Sequence logo | Score | Relative score | Predicted sequence |
| --- | --- | --- | --- | --- | --- | --- |
| ieCtnnb1  (*Mus musculus*) | CREB1 | MA0018.2 | 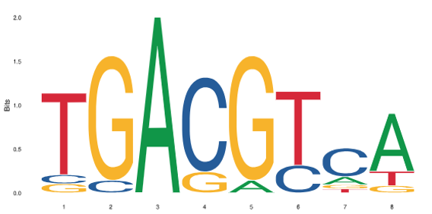 | 9.92 | 0.93 | TGACGTCT |
|  | HNF4𝛼 | MA0114.3 | 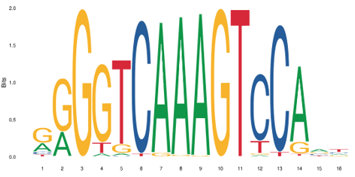 | 16.39 | 0.90 | GGGGGCAAAGTCTATC |
| *Ctnnb1* Promoter  (*Mus musculus*) | CREB1 | MA0018.2 | 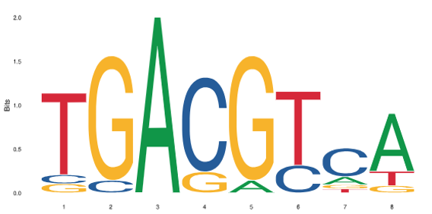 | 8.00 | 0.86 | TGAAGTCA |
|  | HNF4𝛼 | MA0114.3 | 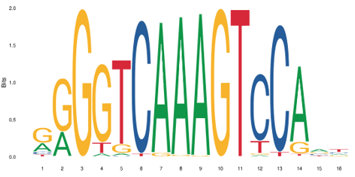 | 5.18 | 0.79 | CGGTTCCAAGTCTGGT |
| ieCTNNB1  (Homo sapiens) | CREB1 | MA0018.5 | 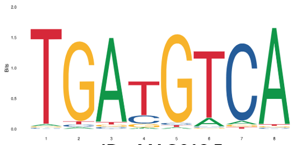 | 14.63 | 0.99 | TGATGTCA |
|  |  | MA0018.4 | 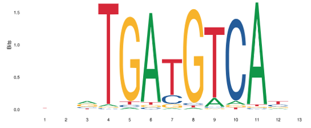 | 14.29 | 0.95 | CTCTGATGTCACC |
|  | HNF4𝛼 | MA0114.2 | 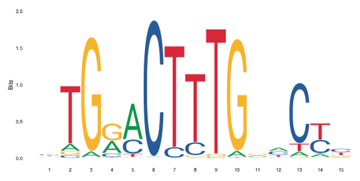 | 10.15 | 0.87 | CTAAACTGTGAACTC |
|  |  | MA0114.5 | 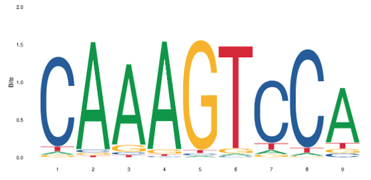 | 9.78 | 0.86 | CAGAGTCCT |
| ieCTNNB1 Promoter  (Homo sapiens) | CREB1 | MA0018.1 | 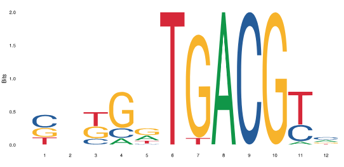 | 10.00 | 0.89 | CGGGGTGACGGC |
|  | HNF4𝛼 | MA0114.2 | 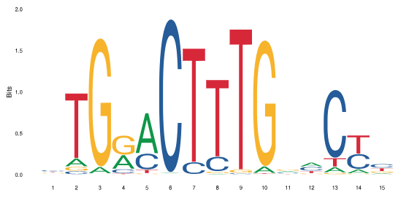 | 14.99 | 0.93 | GAGGACTTTGAACCG |
